# A Cross-Sectional, Randomized Cluster Sample Survey of Household Vulnerability to Extreme Heat among Slum Dwellers in Ahmedabad, India

**DOI:** 10.3390/ijerph10062515

**Published:** 2013-06-18

**Authors:** Kathy V. Tran, Gulrez S. Azhar, Rajesh Nair, Kim Knowlton, Anjali Jaiswal, Perry Sheffield, Dileep Mavalankar, Jeremy Hess

**Affiliations:** 1 Department of Environmental Health, Emory University School of Public Health, Atlanta, GA 30322, USA; E-Mail: kathy.tran@alum.emory.edu; 2 Indian Institute of Public Health, Gandhinagar, Gujarat 380054, India; E-Mails: gsazhar@iiphg.org (G.S.A.); rnair@iiphg.org (R.N.); dmavalankar@iiphg.org (D.M.); 3 Ahmedabad Heat and Climate Study Group, Gandhinagar, Gujarat 380054, India; E-Mails: kknowlton@nrdc.org (K.K.); ajaiswal@nrdc.org (A.J.); perry.sheffield@mssm.edu (P.S.); 4 Natural Resources Defense Council, New York, NY 10011, USA; 5 Icahn School of Medicine at Mount Sinai, New York, NY 10029, USA; 6 Department of Emergency Medicine, Emory University School of Medicine, Atlanta, GA 30322, USA

**Keywords:** heat, climate change, India, vulnerability

## Abstract

Extreme heat is a significant public health concern in India; extreme heat hazards are projected to increase in frequency and severity with climate change. Few of the factors driving population heat vulnerability are documented, though poverty is a presumed risk factor. To facilitate public health preparedness, an assessment of factors affecting vulnerability among slum dwellers was conducted in summer 2011 in Ahmedabad, Gujarat, India. Indicators of heat exposure, susceptibility to heat illness, and adaptive capacity, all of which feed into heat vulnerability, was assessed through a cross-sectional household survey using randomized multistage cluster sampling. Associations between heat-related morbidity and vulnerability factors were identified using multivariate logistic regression with generalized estimating equations to account for clustering effects. Age, preexisting medical conditions, work location, and access to health information and resources were associated with self-reported heat illness. Several of these variables were unique to this study. As sociodemographics, occupational heat exposure, and access to resources were shown to increase vulnerability, future interventions (e.g., health education) might target specific populations among Ahmedabad urban slum dwellers to reduce vulnerability to extreme heat. Surveillance and evaluations of future interventions may also be worthwhile.

## 1. Introduction

India is a rapidly developing country with many climate-sensitive health concerns [1]. The incidence of weather-related illness in India is not known, but historically heat illness has been a significant issue [2]. Temperatures are highest in the summer (March-May), averaging between 30–35 °C in most of the interior. Daily maxima reach 40 °C in many locations and exceed 45 °C in some north and north-west regions [3]. 

Climate change is expected to bring increasingly frequent and severe extreme heat events to the region [4]. Mean annual temperatures across India have been over historical normals (1961–1990) since 1990, with annual increases between 0.1 and 1 °C between 1990 and 2009 [3]. This is consistent with global circulation model projections [3,5]. Major heat waves occurred in 1998 and 2003 in several regions of India. In 1998, temperatures rose to 45.4–47.6 °C in affected areas [6]. In 2003, in central to southern India, temperatures soared to above 50 °C, 9–10 °C above normal in several locations [7]. 

These events had significant public health impacts. From 1978–1999, heat waves of variable lengths claimed thousands of lives [8]. In particular, the major heat waves of 1988, 1998, and 2003 resulted in 1,300 [5], 2,541 [6], and 1,900 [7] deaths, respectively. Due to variable reporting, these statistics are almost certainly significant underestimates [9]. 

As both the health impacts of climate change and effective public health preparedness are place-specific [10], factors affecting heat vulnerability in India are of increasing concern. There are, however, relatively few studies of heat vulnerability specific to South Asian populations available to guide adaptive management.

**Figure 1:**
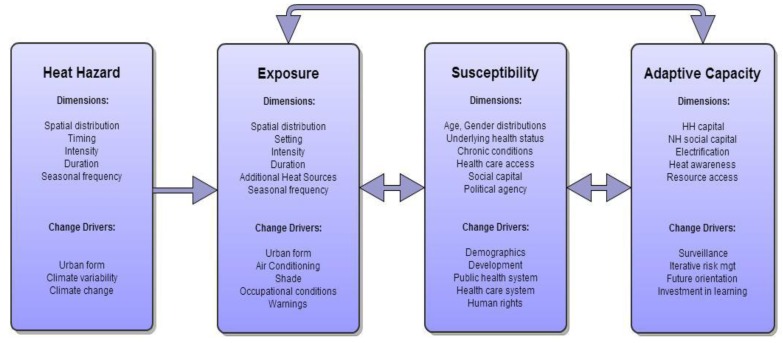
Framework for assessing heat vulnerability: hazard probability, population exposure, susceptibility factors, and adaptive capacity.

Heat vulnerability can be conceptualized as a function of interacting biophysical and socioeconomic determinants that can be broken down into heat hazard probability as well as factors associated with population exposure, susceptibility, and adaptive capacity (Figure 1). Exposure refers to the degree to which the host (e.g., a person, household, neighborhood, or city) is physically exposed to the hazard. Exposure can be affected by hazard factors (e.g., magnitude, persistence, distribution), amplifying factors (e.g., buildings that retain heat, urban heat islands), and protective factors (e.g., air conditioning, exposure avoidance) [11,12,13]. Susceptibility (or sensitivity) relates to the impact of exposure, and is influenced by host characteristics such as demographics (e.g., age, socioeconomic status, social capital) and underlying health status (e.g., obesity, comorbid conditions) [13,14,15,16,17,18,19]. Adaptive capacity is the ability to make protective changes to reduce health burdens, in response to actual or expected hazards [14,15]. In the context of heat as a health hazard, these factors can be influenced and driven by climate variability, urban form, occupational conditions, infrastructure, and interventions that might include warnings, surveillance, and education.

The literature on heat vulnerability in South Asia is underdeveloped; studies have mainly centered on developed countries and mortality after record breaking heat waves [13,15,16,17,18,19]. Work-related heat stress has been studied in a handful of settings in India [19], e.g., outdoors under the sun, in poorly ventilated indoor workspaces, and near furnaces [19,20]. Strenuous activities such as quarrying also amplify heat exposure [20]. Exposure has not been explored in other settings. There is also scant literature on exposure factors such as recent historical shifts in extreme heat hazards; on susceptibility factors such as age [21]; and on adaptive capacity for heat, though there are relevant papers on electricity demand [22] and for other hazards [11,23]. 

The framework in Figure 1 was used to explore vulnerability to extreme heat in Ahmedabad, India, a rapidly growing city of 7.2 million [24] and very high summer temperatures. The city experiences severe heat waves frequently, with one in 2010 and 2012. The 2010 maximum temperature of 46.8 °C set a 40-year temperature record. A randomized, cluster-sampled survey of slum households (as defined by the Indian National Sample Survey Organization [25]) was used to explore associations between heat illness and household vulnerability factors. Exposure, susceptibility, and adaptive capacity factors were determined a priori through literature review and expert opinion. Outcomes were self-reported heat-related illness and heat-related symptoms at the individual level. Exposure factors included geographic location, housing characteristics, and occupational and behavioral factors. Susceptibility components included age, preexisting health status, and socioeconomic factors. Adaptive capacity factors included access to health services and information, coping mechanisms, and societal factors (infrastructure, information, and social capital) [26]. The goal was to gain insight into factors affecting heat vulnerability to facilitate public health preparedness in Ahmedabad, with the potential for generalizing findings to other South Asian cities subject to similar heat hazards.

## 2. Materials and Methods

### 2.1. Study Setting

**Figure 2:**
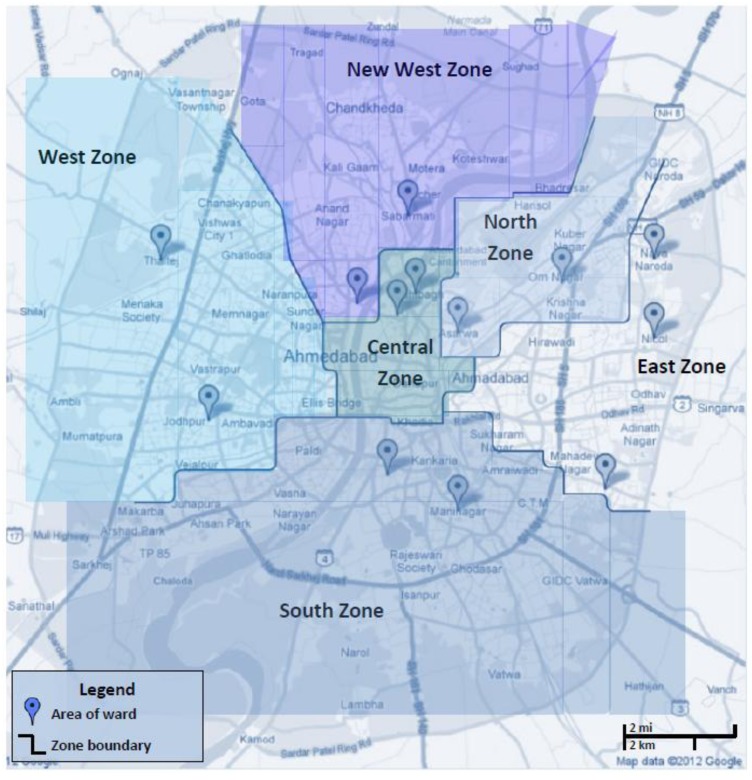
Location of 13 randomly selected wards. Markers represent the central location of the sampled wards as a GPS was not accessible. Two wards per zone were sampled in each of five zones (West, New West, South, Central, North); and three wards were sampled in a sixth zone (East). West zone: Sabarmati, Juna Vadaj; New West zone: Thaltej, Johdpur; South zone: Maninagar, Behrampura; Central zone: Madhupura, Dudeshwar; North zone: Saijpur, Asarwa; East zone: Nikol, New Naroda, Ramol.

Ahmedabad is the largest city of Gujarat state in western India and situated in a sandy, dry area along the banks of Sabarmati River. The city is administratively divided into six zones, each of which is subdivided into wards (Figure 2). Ahmedabad is arid year-round, with summer temperature maxima averaging 45 °C and minima 23 °C. Temperature maxima during summer heat waves have increased recently, e.g., from 45.4 °C in 1998 to 46.8 °C in 2010 [27]. 

Ahmedabad’s slums [25] served as the study population since slum dwellers were hypothesized to be particularly vulnerable based on lack of stable income and basic services (see Supplemental for first-hand observations). Living in densely populated urban slums with poor sanitation, slum dwellers are at high risk for fecal-oral communicable diseases [28]. When they fall ill, few seek treatment because they often do not perceive an illness as serious and/or have financial constraints despite the availability of multiple healthcare services [29]. About 25.8%, or 900,000 Amdavadis in Ahmedabad city, resided in slums as of 2006 [30]. 

### 2.2. Sampling Strategy

**Figure S1:**
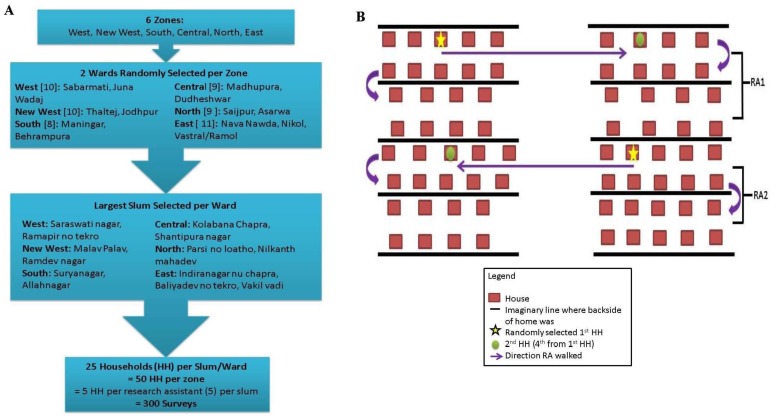
(**A**) Sampling design: Among the 6 city zones, two wards were randomly selected per zone (out of 57). The largest slum was selected (12 and one additional in East zone where boundaries were unclear), where 25 households were surveyed for a total of 25 households per slum and 300 total surveys; (**B**) Household selection method**:** The neighborhood layout varied among slums. An example of one organized by parallel lanes is shown. Research assistants (5) randomly selected one household to begin at in assigned area. After the initial household, they proceeded to every fourth, right or left of the initial.

All six city zones (Central, North, South, East, West, and New West) were sampled by randomly selecting two wards per zone for a total of twelve wards and selecting the largest slum per ward (Figure S1(a)). A 13th ward, not randomly selected, was also sampled because we unknowingly crossed ward boundaries; redistricting occurred in 2010. With the help of community health workers, five research assistants (RAs) were each assigned a random area across each slum to sample from (Figure S1(b)) at all 13 study sites (Figure 2) to prevent potential bias from inherent differences among residents residing in different locations of the settlements. At the starting location, households were randomly selected by each RA, followed by systematic selection of every fourth house thereafter. Twenty-five surveys were completed in each ward (50 per zone).

### 2.3. Data Collection Methods

The survey was drafted in English and translated to Gujarati (available upon request). The RAs read questions and response choices to self-identified heads-of-household in Gujarati. Females were preferred since it was assumed they were the most familiar with their familys’ activities and health histories. However, males were not denied if they were the only ones present or their wives preferred not to respond. Surveys were completed, on average, within 45 min. An author checked all surveys for completeness and errors at the end of each field work day. No incentives were provided (financial or otherwise) to study participants. Despite this, response was relatively high possibly because most women were unemployed within this population and felt comfortable because community health workers were present. An information sheet with tips for reducing risk to heat exposure and preventing heat-related illnesses was given to each household after the interview.

### 2.4. Assessment of Vulnerability Factors

Survey questions were generally close-ended and examined self-reported preexisting health conditions, heat-related illnesses and symptoms, indoor/outdoor heat exposure (home, transit, occupational), behavioral adaptations to heat, sources of weather and health information, and social connectedness. Open-ended responses were not included in analysis due to the variety of responses received. Close-ended survey responses were either dichotomous or categorical. There were both individual and household level survey questions; respondents served as proxy for their household members. Individual level questions included those regarding demographics, health conditions (preexisting and heat-related symptoms and diagnoses), and occupational settings. Most questions regarding behaviors collected information at the household level.

### 2.5. Outcomes and Data Management

Primary outcomes of interest included self-reported heat-related symptoms (HRS) and diagnosed heat-related illnesses (HRI). Respondents reported any HRS and HRI they or their family members ever experienced (see Supplemental). To increase power, symptom and illness options were condensed into a single binary variable where “yes” corresponded to ever experiencing any of those symptoms or illnesses. Heat-related illnesses diagnosed by a healthcare provider were ranked by severity from mild to severe for descriptive purposes only (see Supplemental). An additional composite dichotomous outcome, accounting for those with reported HRS and/or HRI, was also created. 

To assess associations between coping methods and social connectedness and self-reported HRS and HRI, each household was given a score for coping ability and social connectedness (see Supplementary). The coping score was based on the frequency of applying eight coping techniques at the household level. The social connectedness score was developed based on household level indicators of social connectivity and reliance on neighbors in times of need. The coping level and social connectedness score were regarded as categorical for the regression models. 

### 2.6. Statistical Analysis

All analyses were performed using SAS 9.3 (SAS Institute, Inc., Cary, NC, USA). For descriptive purposes, variables were categorized as demographics, exposure, susceptibility, adaptive behaviors, and outcomes, and examined for regional differences between zones. Multiple logistic regression using generalized estimating equations (GEE) to account for clustering effects at the household and slum levels was performed to test the various hypotheses. Under GEE, binomial distribution, logit transformation, and an exchangable covariance matrix (compound symmetry) were utilized. Accounting for possible clustering effects facilitated examination of all outcomes and covariates at the individual level regardless of whether they were collected at individual or household levels. 

Covariates were selected based on statistically significant unadjusted odds ratios (ORs) for each outcome. “Interviewer” was included in all adjusted models as a possible confounder because the beta estimates changed more than 10% for most covariates in unadjusted models. Missing variables, for two covariates, were accounted for in all models. After determining the adjusted model for each outcome, covariates specifically being tested in hypotheses were included in the model regardless of the statistical significance of their unadjusted ORs. Adjusted ORs were also internally validated by creating contingency tables and estimating ORs (unadjusted for clustering) to check for agreement in general direction (less than or greater than 1) of the ORs.

## 3. Results and Discussion

The response rate was 97.7% and a total of 300 households were enrolled. Information was provided for 1,650 individuals in total. Descriptive analysis results are presented below by each component of vulnerability and analysis type. Unless specifically indicated, proportions reported are for all individuals in the sampled households, not just the proxy responder that was interviewed.

### 3.1. Demographics

All zones sampled were demographically similar (Table 1). Most proxy respondents were young females. On average, households had between 5–6 people and about half contained at least one young child and/or elderly person, though proportions varied by zone. The average monthly household income was 6,389 INR (=$142 USD, 2011), slightly above the Gujarat state urban poverty line of 5,708 INR/month (=$127 USD) (household of 6) (India Planning Commission 2012). The distribution was skewed to the right, and median was 5,000 INR (=$111 USD). 56% of working age (15–50) household members and 19% of the elderly were employed. Home ownership rates were 74–88% across all zones. 92% of households reported paying for electricity, with averages just under 300 INR per month.

### 3.2. Heat Illness

Self-reporting of heat-related symptoms (HRS) and illnesses (HRI) ever experienced varied by zone (Table 2). HRS were reported for more than one person per household on average, whereas HRI were less common, ranging from 0.2 to 1.02 per household. An average of 20.1% reported HRS and 11.9% HRI. Most HRI were heat rash, heat edema and heat exhaustion. More severe HRI were relatively rare at 1.3%. When self-reported HRS and HRI were combined, 28.9% of the sample (477) had experienced these outcomes. Of these 477, 50 (10.5%) had experienced both heat-related symptoms and illnesses in their lifetimes. 

### 3.3. Exposure Factors

Potential heat exposure occurred at home, work and in transit (Table S1). Most individuals (63%) were homemakers or unemployed. Almost all respondents (96.7%) reported their homes were warmer than outside during the summer, though only 11.0% go somewhere with air conditioning (AC). 89.3% had an indoor kitchen and 94.1% indicated their home was hotter when cooking. Among households with indoor kitchens, 85.5% kept their windows open during the summer, and 94.1% while cooking. 93.7% used fans at home.

Among employed household members, occupations were widely distributed, and some zones had concentrations of particular occupations. Among the employed, 98.5% worked during the day, 89.7% worked outdoors, and 46.5% worked in the shade. Lastly, 74.3% of respondents primarily walk as a mode of transit.

### 3.4. Susceptibility Factors

Preexisting conditions and limited access to resources influenced susceptibility (Table S2). On average, households had approximately three members with preexisting conditions, though prevalence varied considerably by zone. Infectious diseases (excluding diarrheal) were most commonly reported (22.7%), followed by chronic conditions (17.8%) and diarrheal illnesses (5.7%). Diarrheal diseases were more commonly reported among young children (14.2%) and chronic diseases more commonly among elderly (55.7%), though this varied among the zones. While AC and water access were limited, most respondents (73.7%) denied any specific barriers to accessing AC because they did not routinely think of it as an option, as indicated through conversation. Most reported in-home taps (86.7%) with water access restricted to the morning (93.0%).

### 3.5. Adaptive Factors

Adaptive behaviors included access to healthcare, coping strategies, and social capital (Table S3). It is convenient for 99.0% of households to see a doctor. 77.3% spoke with a health care provider for advice on preventing heat illness, and 46.3% have accessed the health care system for heat-related symptoms. The most common coping strategies were staying indoors (90.0%), drinking plenty of water (90.3%), and wearing head coverage during transit (96.0%). Going to a place with AC (9.7%), avoiding outdoor activities (6.7%), and reducing activity overall (15.3%) were uncommon. Most households heard heat warnings in 2011; 53.3% heard them through others and 46.7% through media, primarily television (results not shown). Regarding social connectedness, 97.3% felt safe in their neighborhoods, 86.0% talked with neighbors often, and 98.3% would call on neighbors during an emergency; 78.7% reported having done so in the past.

**Table 1:** Household demographics of the sampled Ahmedabad slum dwellers across six city zones (household N = 300, individual N = 1,650).

	West	New West	South	Central	North	East	Entire Sample
# HH sampled	50	50	50	50	50	50	300
n (%) F respondent	44 (88.0)	39 (78.0)	47 (94.0)	45 (90.0)	44 (88.0)	45 (90.0)	264 (88.0)
n individuals reported	280	269	283	269	287	262	1,650
Avg. ± SD HH size	5.6 ± 2.3	5.4 ± 1.6	5.7 ± 2.8	5.4 ± 2.0	5.7 ± 1.9	5.2 ± 1.7	5.5 ± 2.1
Avg. ± SD age ^a^	26.4 ± 17.9	26.1 ± 18.6	24.2 ± 17.1	26.6 ± 17.2	27.0 ± 17.7	26.4 ± 17.5	26.1 ± 17.7
n (%) HH with young children and/or elderly	25 (50)	23 (46)	24 (48)	23 (46)	27 (54)	16 (32)	144 (48)
Avg. ± SD HH monthly income	6,180 ± 3,634	6,626 ± 5,902	5,716 ± 4,547	5,686 ± 3,919	6,362 ± 4,570	7,755 ± 6,887	6,389 ± 4,913
Avg. ± SD proportion of HH members employed	0.36 ± 0.17	0.39 ± 0.25	0.35 ± 0.15	0.41 ± 0.21	0.40 ± 0.21	0.35 ± 0.16	0.38 ± 0.20
Avg. ± SD proportion of HH members of working age (16–50) employed	0.55 ± 0.22	0.57 ± 0.25	0.58 ± 0.23	0.56 ± 0.22	0.55 ± 0.19	0.53 ± 0.20	0.56 ± 0.22
n (%) elderly (>60) employed among elderly (n = 70) ^a^	1 (8.0)	3 (20.0)	2 (18.0)	3 (30.0)	3 (25.0)	1 (10.0)	13 (19.0)
% (n) own home	41 (82.0)	40 (80.0)	42 (84.0)	38 (76.0)	40 (88.0)	37 (74.0)	243 (81.0)
Avg. ± SD time at current residence (years)	23.3 ± 15.3	28.9 ± 20.1	22.9 ± 28.7	25.1 ± 16.9	32.6 ± 19.5	15.8 ± 12.3	24.8 ± 20.0
n (%) pay for electricity	48 (96.0)	46 (92.0)	43 (86.0)	44 (88.0)	50 (100)	44 (88.0)	276 (92.0)
Avg. ± SD bi-monthly electric bill (INR)	639.9 ± 526.9	576.4 ± 357.5	433.8 ± 355.0	576.1 ± 407.9	762.8 ± 630.8	522.6 ± 533.0	585.3 ± 486.2

^a^ Among individuals (n differs by zone).

**Table 2:** Prevalence of self-reported outcomes within individuals (N = 1,650) residing in Ahmedabad slums across six city zones within the sampled population.

	West	New West	South	Central	North	East	Entire Sample
n individuals reported	280	269	283	269	287	262	1,650
Avg. ± SD of HH members with heat-related symptoms	1.2 ± 1.11	0.92 ± 1.08	0.92 ± 1.23	0.80 ± 0.83	1.44 ± 1.01	1.36 ± 0.94	1.11 ± 1.06
Avg. ± SD of HH members with heat-related illnesses	0.88 ± 1.08	0.20 ± 0.46	0.86 ± 1.81	0.38 ± 0.70	1.02 ± 0.92	0.85 ± 0.85	0.69 ± 1.09
n (%) who *ever* previously experienced a heat-related symptom	60 (21.4)	46 (17.1)	46 (16.3)	40 (14.9)	72 (25.1)	68 (26.0)	332 (20.1)
n (%) who was *ever* previously diagnosed with:							
Heat stroke	0 (0.0)	0 (0.0)	0 (0.0)	0 (0.0)	0 (0.0)	1 (0.38)	1 (0.06)
Hyperthermia	4 (1.4)	0 (0.0)	7 (2.5)	2 (0.74)	5 (1.7)	1 (0.38)	19 (1.2)
Heat rash/edema/exhaustion	35 (12.5)	6 (2.2)	35 (12.4)	15 (5.6)	45 (15.7)	39 (14.9)	175 (10.6)
None	241 (86.1)	263 (97.8)	241 (85.2)	252 (93.7)	237 (82.6)	221 (84.6)	1,755 (88.2)
n (%) with composite outcome (ever had a heat-related symptom or heat illness diagnosis)	84 (30.0)	51 (19.0)	79 (27.9)	52 (19.3)	109 (38.0)	102 (38.9)	477 (28.9)

### 3.6. Univariate Analyses

Univariate associations between certain demographic, exposure, susceptibility, and adaptive factors and the three outcomes are presented in Table S4. Significance of these associations varied between the three outcomes. Covariates that significantly increased unadjusted odds ratios included: old age (>60), increasing household income (by every 100 INR), working in the sun, having indoor kitchens, walking (as primary mode of transit), all categories of pre-existing conditions, having barriers to accessing AC, previously visiting a doctor for HRI, seeking heat-health information in the past, and exposure to heat warnings by word of mouth. Significant protective covariates included: young ages (<5), increasing electricity spending (by every 100 INR) decreased unadjusted odds, AC and fan usage, and drinking tap or public water (compared to bottled water). Unadjusted ORs for several coping mechanisms were individually significant with the outcomes; some were protective, while some increased odds. Individual social connectedness variables did not show many significant associations, though higher levels of social connectedness were associated with decreased unadjusted odds of all outcomes.

### 3.7. Multivariate Analyses

Multivariate analyses were used to test specific hypotheses while controlling for factors identified as significant in the univariate analyses (Table 3, Table 4 and Table S5). 

Age, work location, preexisting conditions, water resources, and information access all resulted in significant adjusted associations. Adjusted odds of HRS and the composite outcome significantly increased with age (results for age as a continuous variable not shown). For children <5 years old, the odds of HRS were 0.13 (0.04, 0.47) while the odds of the composite outcome were 0.41 (0.24, 0.70) (Table 4). Among those >60 years old, the odds of HRS were 1.96 (1.17, 3.28) and odds of the composite outcome were 1.90 (1.07, 3.37). Chronic and infectious preexisting conditions increased adjusted odds of all three outcomes while diarrheal conditions only increased odds for HRI. The odds of HRS among those who used a public source (tap or borehole) were 0.41 (0.19, 0.90) compared with those who purchased bottled drinking water (Table 3). 

No specific occupations showed significant associations. However, working in the sun was a risk factor, with an adjusted OR of 2.27 (1.31, 3.94) for HRS and 1.86 (1.09, 3.16) for the composite outcome (Table 4). 

Significant associations were observed between health information access and the outcomes. Notable findings included the observation that those who had *not* sought any information on heat-related illness had an adjusted OR of 11.18 (2.75, 45.38) for HRI and 2.54 (1.20, 5.49) for the composite outcome (Table S5). Worrying about getting sick from heat was associated with an adjusted OR of 2.84 (1.25, 6.45) for HRS and 2.22 (1.04, 4.75) for the composite outcome. Having seen a doctor for HRI also had an adjusted OR of 2.77 (2.13, 3.57) for the composite outcome.

Results on social connectedness did not show a consistent relationship in either univariate or multivariate analyses.

## 4. Discussion

In this sample of Ahmedabad slum dwellers, age, work location, preexisting medical conditions, drinking water sources, and information access were associated with vulnerability to heat as measured by self-reported heat illness symptoms and diagnoses. Several results were consistent with prior literature while some were unexpected findings.

Age, chronic and diarrheal diseases, and working conditions all increased the odds of the outcomes, as expected. However, unlike elderly age, young age (<5) was protective. Caregivers might have been more vigilant in caring for the young leading to reduced heat exposure. Working outdoors directly under the sun also was associated with increased odds of all outcomes, presumably as a result of increased exposure. In addition, seeking information on HRI reduce the odds of the outcomes. Respondents possibly took more preventative measures if they were familiar with the health effects of heat.

There were also some unexpected findings. Somewhat counterintuitive, increasing monthly household income (by 100 INR) increased odds of heat-related symptoms in univariate analysis. Occupational exposure, occupational illness, increased access to doctors, or educational awareness of heat illness may have confounded this relationship. Most of these potential confounders were included in the adjusted multivariate model, where this relationship became insignificant.

While high risk occupational groups have been identified, occupation was insignificant here. This was likely related to statistical power: variations between the occupational groups relative to the outcomes were too sparse, so an association was undetectable even when categories were merged. Conversely, a relationship between infectious diseases (non-diarrheal) and heat-related symptoms or illness has not been previously demonstrated. The novelty of the finding likely reflects the relatively large burden of infectious disease in India compared with developed country settings, where most heat-health research has been conducted. Causality is unclear. It is possible that infectious disease increases heat exposure or susceptibility to illness, or decreases a host’s coping range. Alternatively, the association may largely reflect seasonal co-variance of infectious disease and heat illness, given that endemic diseases in India (e.g., malaria, dengue fever, chikungunya) increase during the summer months. This may also be driven by overall vulnerability to both sets of illnesses in this population since the time course of the preexisting conditions relative to heat illness outcomes was not provided. 

**Table 3:** Covariates included in final model for each heat-related outcome based on significance at the α = 0.05 level (heat-related symptoms, heat-related illnesses, and composite outcome).

	HRS	HRI	HRI + HRS
Age			
Young (<5)	0.13 (0.04, 0.45)	--	0.45 (0.26, 0.75)
Elderly (>60)	1.94 (1.16, 3.25)	--	1.85 (1.05, 3.25)
All other ages (5 ≤ age ≤ 60) (ref)			
Work location:			
Sun	2.22 (1.32, 3.73)	--	1.78 (1.08, 2.93)
Mixed	1.06 (0.63, 1.78)	--	1.10 (0.66, 1.82)
Shade (ref)			
Chronic preexisting condition	3.41 (2.52, 4.61)	1.67 (1.11, 2.52)	2.45 (1.81, 3.30)
Diarrheal preexisting condition	--	3.31 (1.73, 6.32)	--
Infectious preexisting condition	1.58 (1.13, 2.21)	2.84 (1.88, 4.31)	1.81 (1.33, 2.47)
Main drinking water source:			
In-home tap	0.51 (0.26, 0.99)	--	--
Public (tap/borehole)	0.41 (0.19, 0.90)	--	--
From neighbor	0.84 (0.27, 2.58)	--	--
Purchased (bottled or 50 L jugs) (ref)			
Visited a doctor for heat-related illness before	--	--	2.94 (2.22, 3.85)
Had NOT sought heat-related morbidity info before	--	4.58 (1.41, 14.88)	--
Worried about getting sick from heat	2.78 (1.21, 6.35)	--	2.14 (1.03, 4.49)
Social Connectedness Score*:*			
0–1	2.73 (1.33, 5.64)	0.82 (0.31, 2.15)	2.07 (1.10, 3.88)
2	0.72 (0.45, 1.13)	0.42 (0.21, 0.85)	0.70 (0.46, 1.08)
3 (ref)			

**Table 4:** Association between the heat-related outcomes and age, occupation, and preexisting conditions (adjusted ORs and 95% confidence intervals).

	HRS	HRI	HRI + HRS
*Age ^a^*			
Young (<5)	0.13 * (0.04, 0.47)	0.90 (0.53, 1.52)	0.41 * (0.24, 0.72)
Elderly (>60)	1.96 * (1.17, 3.28)	0.98 (0.40, 2.40)	1.90 * (1.07, 3.37)
All other ages (5 ≤ age ≤ 60) (ref)			
*Occupation ^a^:*			
Manual labor	0.91 (0.45, 1.83)	0.69 (0.43, 1.10)	0.76 (0.36, 1.61)
Service/Office/teacher	0.80 (0.40, 1.62)	0.78 (0.42, 1.46)	0.50 (0.23, 1.09)
Sales/Artisan	1.05 (0.54, 2.08)	0.92 (0.52, 1.66)	0.92 (0.44, 1.90)
None (ref)			
Work location:			
Sun	2.27 * (1.31, 3.94)	--	1.86 * (1.09, 3.16)
Mixed	1.09 (0.64, 1.85)	--	1.10 (0.66, 1.84)
Shade (ref)			
*Chronic preexisting condition ^a^*	3.44 * (2.54, 4.67)	1.71 * (1.13, 2.59)	2.50 * (1.86, 3.36)
*Diarrheal preexisting condition ^a^*	0.93 (0.52, 1.66)	3.19 * (1.68, 6.07)	1.63 (0.96, 2.76)
*Infectious preexisting condition ^a^*	1.59 * (1.13, 2.23)	2.83 * (1.86, 4.29)	1.82 * (1.34, 2.47)
Main drinking water source:			
In-home tap	0.51 (0.26, 1.01)	--	--
Public (tap/bore hole)	0.42 * (0.19, 0.91)	--	--
From neighbor	0.83 (0.27, 2.56)	--	--
Purchased (bottled/50 L jug) (ref)			
Visited a doctor for heat-related illness before	--	--	2.85 * (2.22, 3.85)
Had NOT sought heat-related morbidity info	--	4.75 * (1.45, 15.55)	--
Worried about getting sick from heat	2.79 * (1.23, 6.31)	--	2.19 * (1.05, 4.54)
*Social Connectedness Score ^a^*			
0–1	2.77 * (1.33, 5.76)	0.84 (0.32, 2.20)	2.19 * (1.17, 4.10)
2	0.71 (0.45, 1.13)	0.43 * (0.21, 0.87)	0.71 (0.46, 1.09)
3 (ref)			

^a^ Covariates were tested in the hypothesis; * statistically significant at α = 0.05.

Interestingly, worrying about heat illnesses and previously visiting a doctor for HRI were not protective, and social connectedness had mixed results. Those who reported being worried and previously visited doctors for HRI were probably more likely to report experiences of heat symptoms and illnesses, shifting the direction of the association. While low social connectedness was generally associated with increased odds of the outcomes and higher social connectedness with decreased odds, the significance of these relationships was not consistent across all outcomes or analyses. Confounding or interactions between covariates, not explored here due to data limitations, might explain these behavioral anomalies. Alternatively, there may be limitations related to the ways in which identifying HRI and social connectedness was assessed. The survey did not query whether respondents visited doctors for HRI because they recognized symptoms or were diagnosed after seeking care for another ailment. Furthermore, to the authors’ knowledge, there are no validated instruments for assessing social capital and social connectedness in India. Additional research is required to explore the recognition of HRI among patients and doctors, and the likely complex ways in which social dynamics may affect health outcomes during periods of high heat exposure.

### 4.1. Limitations

The main limitation is the lack of exposure information, which precluded calculations of relative risks and evaluation of how specific vulnerability factors may affect risk of heat illness among the exposed.

As a cross-sectional survey-based study, some design-related limitations may threaten the study’s internal validity, including reporting and recall bias, interviewer bias, and biases in the survey instrument. Reporting and recall biases are perhaps the most significant concerns.

Reliance on head-of-households to serve as proxies for other household members likely resulted in under-reporting of both symptoms and illnesses, and potential misclassification of exposure information, particularly for groups working outside the home. This probably caused underestimates of effects. Summer 2011 was also not as hot as previous years so behavioral patterns and symptoms likely differed, potentially resulting in lack of associations with more adaptive behaviors.

Additionally, findings captured patterns of exposures and outcomes from one point in time. As subjects were not queried on the dates of their symptoms, the magnitude of this potential bias is difficult to determine. Overall, health conditions were likely underreported, which introduced a systematic bias resulting in underestimation of observed associations.

Interviewers and the survey posed additional limitations. While RAs were trained in survey administration and observed data collection in the field, they were not academically trained in health or social sciences and lacked research experience. Consequently, they may have unintentionally biased certain responses by non-standardized question delivery. Response patterns were detected among different RAs. In such cases, responses were excluded (10% of questions). Besides reducing statistical power by reducing the number of valid responses, however, it is difficult to assess how this may have impacted the results. To account for possible confounding, an interviewer term was included in all multivariate models. 

Given that we were developing a tool to assess exposures and outcomes not previously evaluated in India, the survey instrument possibly did not perform as expected despite efforts to reduce potential error. Respondents may have better understood certain survey terminology, especially medical terms, if they were replaced by regional slang, though this might have invited other issues related to imprecision. It is difficult to speculate as to the impact of these potential biases on study results.

Lastly, results may not be generalizable due to sampling procedures and analysis. Although clustered multistage sampling was conducted, weights could not be assigned for the strata (zones) and sampling units (ward, slum, household) since information for population weights at these levels was unavailable. GEE was sufficient to estimate adjusted ORs only for the sampled population given this limitation.

### 4.2. Implications for Future Research and Interventions

Findings here can serve as a baseline for patterns of heat-related morbidity and vulnerability factors among urban slum dwellers in Ahmedabad, India. Priority should be placed on efforts to establish associations between heat exposure and valid indicators of health outcomes, ideally in occupational and domestic settings, for future studies. Temperature can be monitored in specific slum settlements to compare ambient temperatures from central monitors to regional monitors, and to link with morbidity and mortality data. Actual heat exposures can then be better understood in relation to temporal and spatial morbidity and mortality patterns. There is also a growing need for intervention research describing heat-health interventions; factors affecting intervention deployment, implementation, and reproduction; and intervention impacts and cost-effectiveness. It is important to pursue such research in low-resource settings, as the context may significantly affect both implementation and efficacy.

Combined with existing knowledge regarding public health prevention of heat illness, potential interventions that might reduce vulnerability to heat illness among Ahmedabad slum residents (and potentially similar South Asian populations) were identified. These include:

(1) Heat-health education of vulnerable populations via established community health workers and community leaders, whom slum dwellers frequently interact with and trust. Televised heat warnings and tips might also help, as 78% reported television as their primary source for weather information.
(2) Increasing awareness of heat-related illnesses among health careproviders to help them recognize signs and symptoms and provide anticipatory counseling to patients at risk. Educating providers and vulnerable populations can lead to early detection.
(3) Targeting interventions to reduce risk among the elderly and in specific non-shaded work settings.
(4) Providing constant piped water to slum settlements since greater access to water can prevent dehydration and allow for cool showers. Water from pipelines and public sources e.g., bore holes were shown to be protective, suggesting greater access compared to those who purchased water.
(5) Establishing a heat illness and mortality tracking system to track intervention efficacy.

## 5. Conclusions

This cross-sectional study used randomized cluster sampling of slum dwellers in Ahmedabad, India to evaluate factors potentially associated with vulnerability to heat, a prevalent concern that is expected to worsen with climate change. Symptoms of heat illness were reported among one fifth of respondents, though severe heat illness was reported among approximately 1%. Age over 60 years, having preexisting medical conditions, outdoor work location, and limited access to water or information resources were found to increase the odds of heat-related symptoms and illnesses among urban slum dwellers. Based on these findings and the heat-health literature, reducing this population’s vulnerability might be accomplished by: working with community health workers and leaders to disseminate heat-health information; educating health care providers to increase diagnosis, treatment, and anticipatory guidance to patients; and establishing a heat-health effects tracking system. Future research should assess exposure-outcome associations and focus on intervention implementation and evaluation.

## Abbreviations

RA: research assistant
HRS: heat-related symptoms
HRI: heat-related illness
HH: household
INR: India rupee
SD: standard deviation
AC: air conditioning

## Appendix

### Variable Definitions and Recoding Methods

Heat-related symptoms and illnesses: Symptoms included small blisters or pimples, dry mouth, fatigue, leg cramps, heavy sweating, intense thirst, rapid heartbeat, headache, and leg swelling. Because having one or several symptoms could result in heat-related illnesses of varying degrees, responses were combined into a single binary variable where “yes” corresponded to ever experiencing any of those symptoms before and “no” implied never experiencing those symptoms before. Heat-related illnesses diagnosed by a healthcare provider were ranked by severity from mild, moderate to severe for descriptive purposes. Mild diagnoses included heat rash, edema, and exhaustion. Moderate diagnoses included hyperthermia. Lastly, severe diagnoses included heat stroke.

Household coping strategies: Coping strategies included staying indoors, drinking plenty of water, seeking shade, wearing light clothing, wearing a hat/covering head, going to an air conditioned place, reducing activity, taking cool showers, and avoiding outdoor activity. Observations were given a score of 2 for each coping mechanism where the response was “most of the time,” 1 for “sometimes,” and 0 for “rarely/none.” Scores were then categorized into three coping ability levels: low (0–5), moderate (6–9), and high (10–16). The range of the index was from 0 to 16.

Social connectedness: These behaviors included: feeling safe in neighborhood because of positive/neutral relations within neighborhood; knowing most of their neighbors and talking to them often; nearest person they would call in an emergency; and neighbors checking on each other during heat waves and/or emergencies. A score of 1 was given to responses of “yes” for those behaviors and 0 for “no.” A score of 1 was also given to “in the neighborhood” and 0 for “no one” or “other” for nearest person respondent would call in an emergency. Scores were categorized as low (0–1), moderate (2), and high (3, reference).

### Description of Slum Settlements

Geographically, the sampled slum communities varied in layout and composition. Some were laid out in a grid while others had been more haphazardly developed. Homes were one or two stories, constructed of concrete or brick, and had roofs of sheet metal, asbestos, or concrete. Most homes had two windows on the front side and no doors or windows in back. Some homes had an enclosed front yard. Most neighborhoods lacked paved roads, improved sewage systems, and latrines, though a number had piped water. Water service was intermittent. Most communities lacked vegetation, and trees were usually located at the perimeters of the neighborhoods. Outside space was limited and typically filled with drying laundry. Free-roaming livestock included cattle and goats.

**Table S1:** Individual (N = 1,650) and household (N = 300) exposure factors among residents of Ahmedabad slums across six city zones.

% (n)	West	New West	South	Central	North	East	Entire Sample
N = 1,650 reported individuals							
Occupation groups	N = 280	N = 269	N = 283	N = 269	N = 287	N = 262	N = 1,650
Homemaker/None	65.7 (184)	61.0 (164)	66.4 (188)	59.9 (161)	62.4 (179)	64.9 (170)	63.4 (1046)
Physical labor	11.4 (32)	11.2 (30)	7.8 (22)	13.0 (35)	13.2 (38)	9.9 (26)	11.1 (183)
Service	13.2 (37)	10.8 (29)	7.4 (21)	11.2 (30)	7.0 (20)	6.1 (16)	9.3 (153)
Office/Teacher	2.5 (7)	1.1 (3)	0.4 (1)	1.9 (5)	0.7 (2)	4.6 (12)	1.8 (30)
Factory/Manufacturing	0.7 (2)	0.7 (2)	3.2 (9)	6.0 (16)	9.4 (27)	9.9 (26)	4.9 (82)
Sales	5.0 (14)	8.6 (23)	3.5 (10)	6.3 (17)	5.9 (17)	1.9 (5)	5.2 (86)
Artisan	1.4 (4)	6.7 (18)	11.3 (32)	1.9 (5)	1.4 (4)	2.7 (7)	4.2 (70)
Work outdoors in summer ^a^	92.7 (89)	77.3 (75)	72.8 (67)	98.2 (106)	97.3 (106)	97.8 (90)	89.7 (533)
Work in the daytime ^a^	97.9 (94)	96.9 (94)	97.8 (90)	99.1 (107)	99.1 (108)	100 (92)	98.5 (585)
Location of work ^a^							
Sun	22.9 (22)	16.5 (16)	14.1 (13)	20.3 (22)	6.4 (7)	10.9 (10)	15.2 (90)
Shade	33.3 (32)	41.2 (40)	62.0 (57)	36.1 (39)	54.1 (59)	53.3 (59)	46.5 (276)
Mix	43.8 (42)	42.3 (41)	23.9 (22)	43.5 (47)	39.5 (43)	35.9 (33)	38.4 (228)
N = 300							
Modes of transit							
Walking	68.0 (34)	74.0 (37)	76.0 (38)	74.0 (37)	78.0 (39)	76.0 (38)	74.3 (223)
Bus	14.0 (7)	16.0 (8)	6.0 (3)	18.0 (9)	14.0 (7)	8.0 (4)	12.7 (38)
Home warmer inside than outside during summer ^b^	87.8 (43)	94.0 (47)	100 (50)	98.0 (49)	100 (50)	100 (50)	96.7 (289)
Indoor kitchen	100 (50)	64.0 (32)	86.0 (43)	92.0 (46)	100 (50)	94.0 (47)	89.3 (268)
Inside home warmer when cooking ^b^	84.0 (42)	78.8 (26)	100 (43)	97.8 (45)	100 (50)	100 (47)	94.1 (253)
Open window when cooking ^b^	92.7 (38)	92.9 (26)	91.4 (32)	100 (38)	100 (44)	100 (43)	96.5 (221)
Keep window open in summer ^b^	81.3 (39)	83.3 (35)	82.2 (37)	88.6 (39)	85.4 (41)	91.8 (45)	85.5 (236)
Primary cooling method: electric fan	90.0 (45)	84.0 (42)	98.0 (49)	96.0 (48)	100 (50)	94.0 (47)	93.7 (281)
Do not go to place with A/C	78.0 (39)	90.0 (45)	94.0 (47)	98.0 (94)	90.0 (45)	84.0 (42)	89.0 (267)

^a^ Among employed (n = 594); ^b^ Missing data.

**Table S2:** Individual (N = 1,650) and household (N = 300) susceptibility factors among residents of Ahmedabad slums across six city zones.

% (n) ^a^	West	New West	South	Central	North	East	Entire Sample
Preexisting chronic condition(s) ^b^	20.0 (56)	7.8 (21)	15.9 (45)	10.8 (29)	27.8 (78)	24.8 (65)	17.8 (294)
Preexisting infectious disease(s) ^b^	24.6 (69)	1.5 (4)	17.3 (49)	18.2 (49)	30.3 (87)	44.7 (117)	22.7 (375)
Preexisting diarrheal disease(s) ^b^	6.1 (17)	2.2 (6)	5.3 (15)	4.1 (11)	8.4 (24)	8.0 (21)	5.7 (94)
Avg. ± SD of HH members with preexisting conditions ^b^	2.84 ± 2.21	1.14 ± 1.40	2.38 ± 1.23	2.00 ± 1.65	3.54 ± 1.85	3.58 ± 1.67	2.58 ± 1.89
Young children with chronic preexisting condition(s) among young children (n = 148) ^b^	6.7 (2)	0.0 (0)	7.7 (2)	0.0 (0)	7.7 (2)	10.0 (2)	5.4 (8)
Young children with diarrheal preexisting condition(s) among young children (n = 148) ^b^	16.7 (5)	12.0 (3)	7.7 (2)	0.0 (0)	30.8 (8)	15.0 (3)	14.2 (21)
Elderly with chronic preexisting condition(s) among elderly (n = 70) ^b^	50.0 (6)	33.3 (5)	63.6 (7)	30.0 (3)	91.7 (11)	70.0 (7)	55.7 (39)
Elderly with diarrheal preexisting condition(s) among elderly (n = 70) ^b^	8.3 (1)	0.0 (0)	18.2 (2)	20.0 (2)	0.0 (0)	10.0 (1)	8.6 (6)
A/C access prevented by:							
Nothing/Don't want to go	54.0 (27)	82.0 (41)	78.0 (39)	92.0 (46)	68.0 (34)	68.0 (34)	73.7 (221)
Time of day	8.0 (4)	8.0 (4)	4.0 (2)	0.0 (0)	6.0 (3)	8.0 (4)	5.7 (17)
Disability or Elderly/young at home	10.0 (5)	0.0 (0)	4.0 (2)	2.0 (1)	20.0 (10)	12.0 (6)	8.0 (24)
Distance	20.0 (10)	8.0 (4)	2.0 (1)	0.0 (0)	6.0 (3)	8.0 (4)	7.3 (22)
Safety	8.0 (4)	0.0 (0)	0.0 (0)	0.0 (0)	0.0 (0)	2.0 (1)	1.7 (5)
Financial problem	0.0 (0)	2.0 (1)	12.0 (6)	6.0 (3)	0.0 (0)	2.0 (1)	3.7 (11)
Main water source is in-home tap	70.0 (35)	90.0 (45)	80.0 (40)	92.0 (46)	94.0 (47)	94.0 (47)	86.7 (260)
Water source only available in the morning	100 (46)	97.6 (41)	94.0 (47)	100 (50)	100 (50)	65.2 (30)	93.0 (264)

^a^ Unless otherwise noted as Avg ± SD; ^b^ Among individuals (n differs by zone).

**Table S3:** Household (N = 300) adaptive behaviors among residents of Ahmedabad slums across six city zones.

% (n)	West	New West	South	Central	North	East	Entire Sample
Seeing a Dr. is convenient	98.0 (49)	98.0 (49)	98.0 (49)	100 (50)	100 (50)	100 (50)	99.0 (297)
Visited a Dr. for HRI	56.0 (28)	20.0 (10)	42.0 (21)	28.0 (14)	74.0 (37)	58.0 (29)	46.3 (139)
Coping methods and frequency of application:							
Stays indoors **most of the time**	98.0 (49)	86.0 (43)	90.0 (45)	96.0 (48)	90.0 (45)	92.0 (46)	92.0 (276)
Drinks lots of water **most of the time**	94.0 (47)	96.0 (48)	92.0 (46)	88.0 (44)	88.0 (44)	84.0 (42)	90.3 (271)
Seeks shade **most of the time**	44.0 (22)	74.0 (37)	50.0 (25)	58.0 (29)	50.0 (25)	50.0 (25)	54.3 (163)
Wears light clothing **most of the time**	58.0 (29)	42.0 (21)	40.0 (20)	42.0 (21)	34.0 (17)	44.0 (22)	43.3 (130)
Takes cool showers **most of the time**	92.0 (46)	82.0 (41)	82.0 (41)	86.0 (43)	90.0 (45)	86.0 (43)	86.3 (259)
**Rarely** goes to place with AC	80.0 (40)	88.0 (44)	94.0 (47)	98.0 (49)	92.0 (46)	90.0 (45)	90.3 (271)
**Rarely** reduces activity	68.0 (34)	90.0 (45)	100 (50)	96.0 (48)	80.0 (40)	74.0 (37)	84.7 (254)
**Rarely** avoids outdoor activity	80.0 (40)	84.0 (42)	100 (50)	100 (50)	98.0 (49)	98.0 (49)	93.3 (280)
Protect themselves in-transit	100 (49)	94.0 (47)	92.0 (46)	92.0 (46)	98.0 (49)	100 (50)	96.0 (287)
Heard an excess heat warning this summer thru ^a^:							
People	54.2 (26)	62.5 (25)	48.9 (23)	44.9 (22)	50.0 (23)	60.9 (28)	53.3 (147)
Media Sources	45.8 (22)	37.5 (15)	51.1 (24)	55.1 (27)	50.0 (23)	39.1 (18)	46.7 (129)
Sought heat-related illness info if they were worried about getting sick from exposure ^a^	82.0 (41)	54.0 (27)	84.0 (42)	96.0 (48)	98.0 (49)	92.0 (42)	84.3 (253)
Sought or would look for heat-related illness info thru media sources	95.7 (45)	86.5 (32)	88.9 (40)	95.8 (46)	89.6 (43)	87.5 (42)	90.8 (248)
Previously talked about preventing HRI with:							
Healthcare professional	70.0 (35)	64.0 (32)	84.0 (42)	88.0 (44)	78.0 (39)	80.0 (40)	77.3 (232)
Commmunity	14.0 (7)	4.0 (2)	0.0 (0)	0.0 (0)	4.0 (2)	2.0 (1)	4.0 (12)
No one	16.0 (8)	32.0 (16)	16.0 (8)	12.0 (6)	18.0 (9)	18.0 (9)	18.7 (56)
Feel safe in neighborhood and have positive/neutral relations with neighbors	96.0 (48)	90.0 (45)	100 (50)	100 (50)	98.0 (49)	100 (50)	97.3 (292)
Know most neighbors and talk to them often	86.0 (43)	74.0 (37)	98.0 (49)	98.0 (49)	78.0 (39)	82.0 (41)	86.0 (258)
Would call on someone in their neighborhood for an emergency	94.0 (47)	96.0 (48)	100 (50)	100 (50)	100 (50)	100 (50)	98.3 (295)
They/their neighbors have helped each other during an emergency and/or heat wave	76.0 (38)	74.0 (37)	76.0 (38)	78.0 (39)	82.0 (41)	86.0 (43)	78.7 (236)

^a^ Missing data.

**Table S4:** Association between heat-related outcomes and candidate covariates (unadjusted ORs and 95% confidence intervals).

	HRS	HRI	HRI + HRS
**Demographics**			
Age			
Young (<5)	0.10 * (0.031, 0.34)	1.02 (0.60, 1.72)	0.42 * (0.26, 0.67)
Elderly (>60)	2.80 * (1.74, 4.49)	1.30 (0.61, 2.77)	2.26 * (1.44, 3.55)
All other ages (5 ≤ age ≤ 60) (ref)			
Monthly HH income (increase in 100 INR)	1.00 * (0.99, 1.00)	1.00 (0.99, 1.00)	1.00 (0.99, 1.00)
Bi-monthly electricity bill (increase in 100 INR)	0.96 * (0.94, 0.99)	0.98 (0.95, 1.02)	0.96 * (0.94, 0.99)
**Exposure factors**			
Occupational category			
Manual labor	1.40 (0.99, 1.96)	0.69 (0.44, 1.10)	1.16 (0.86, 1.56)
Service/Office/teacher	1.18 (0.79, 1.77)	0.82 (0.45, 1.48)	0.89 (0.62, 1.29)
Sales/Artisan	1.09 (0.72, 1.65)	0.80 (0.46, 1.38)	1.01 (0.69, 1.47)
Homemaker/none (ref)			
Work outdoor in summer	0.85 (0.44, 1.66)	0.71 (0.34, 1.47)	0.78 (0.44, 1.38)
Location of work			
Sun	2.67 * (1.65, 4.31)	1.01 (0.49, 2.06)	1.95 * (1.26, 3.02)
Mixed	1.02 (0.64, 1.63)	0.61 (0.33, 1.15)	0.89 (0.59, 1.35)
Shade (ref)			
Kitchen located inside	2.55 * (1.61, 4.06)	2.09 (0.92, 4.74)	2.71 * (1.71, 4.29)
Transit: Walking *vs.* Auto rickshaw (ref)	2.49 * (1.58, 3.92)	1.98 (0.48, 8.05)	2.59 * (1.36, 4.95)
Cooling method			
A/C or air cooler	0.15 * (0.053, 0.44)	--	0.11 * (0.034, 0.36)
Electric fan	0.54 * (0.34, 0.86)	--	0.69 (0.37, 1.27)
Other	0.88 (0.19, 4.04)	--	0.82 (0.15, 4.36)
None (ref)			
**Susceptibility factors**			
Chronic preexisting condition	3.37 * (2.57, 4.43)	1.52 * (1.02, 2.26)	2.52 * (1.95, 3.26)
Diarrheal preexisting condition	1.19 (0.69, 2.07)	3.25 * (1.78, 5.93)	2.05 * (1.34, 3.13)
Infectious preexisting condition	1.51 * (1.12, 2.03)	2.75 * (1.88, 4.02)	2.05 * (1.58, 2.67)
Barrier to A/C *vs.* Nothing/Don’t want to	1.42 * (1.07, 1.89)	2.40 * (1.65, 3.51)	1.90 * (1.43, 2.53)
Main source of drinking water			
In-home tap	0.51 * (0.28, 0.92)	0.71 (0.35, 1.42)	0.41 * (0.20, 0.84)
Public	0.43 * (0.21, 0.89)	0.85 (0.30, 2.42)	0.42 * (0.18, 0.97)
Purchased (bottled/50L jugs) (ref)			
Time of day get water			
Morning	0.94 (0.44, 2.03)	--	1.52 (0.71, 3.26)
Noon	1.58 (0.67, 3.75)	--	3.49 * (1.37, 8.88)
Evening (ref)			
**Adaptive Behaviors**			
*Access to information:*			
Visited a doctor for heat-related illness before	1.64 * (1.45, 2.17)	--	3.85 * (2.86, 5.00)
Heat warning source this past summer: People *vs.* Media (ref)	2.10 * (1.56, 2.81)	0.91 (0.61, 1.36)	1.59 * (1.19, 2.13)
Heat-related morbidity source where they have looked/would look			
Media	0.76 (0.46, 1.25)	1.61 (0.85, 3.05)	1.10 (0.67, 1.81)
Don't Know	0.77 (0.25, 2.32)	0.81 (0.10, 6.40)	0.95 (0.23, 4.01)
Community (ref)			
Person talked to about heat-related morbidity			
No one	0.59 * (0.41, 0.83)	0.67 (0.38, 1.18)	0.57 * (0.40, 0.82)
Community	1.12 (0.58, 2.16)	0.93 (0.37, 2.33)	1.13 (0.57, 2.22)
Professional (ref)			
Worried about getting sick from heat	2.65 * (1.03, 6.83)	--	2.93 * (1.02, 8.48)
Have sought heat-related morbidity info before	2.01 * (1.36, 2.97)	6.31 * (2.20, 18.12)	2.88 * (1.95, 4.26)
*Coping methods frequency:* Most of the time *vs.* Sometimes/Rarely (ref)			
Stay indoors	1.31 (0.76, 2.25)	0.72 (0.28, 1.87)	0.95 (0.53, 1.71)
Drink plenty of water	1.62 (0.94, 2.79)	1.46 (0.62, 3.41)	1.76 * (1.03, 3.01)
Seek shade/tree	0.80 (0.61, 1.06)	0.74 (0.50, 1.09)	0.71 * (0.54, 0.94)
Wear light clothing	0.56 * (0.43, 0.72)	1.10 (0.75, 1.61)	0.68 * (0.52, 0.89)
Wear hat/cover head	0.72 * (0.53, 0.96)	0.92 (0.59, 1.44)	0.78 (0.57, 1.05)
Go to A/C location	0.39 (0.05, 2.85)	--	0.25 (0.035, 1.80)
Reduce activity	1.25 (0.86, 1.83)	0.72 (0.38, 1.35)	1.03 (0.67, 1.57)
Take cool showers	2.09 * (1.31, 3.34)	5.28 * (1.82, 15.29)	2.86 * (1.79, 4.56)
Avoid outdoor activity	0.54 (0.28, 1.02)	0.59 (0.14, 2.44)	0.50 * (0.27, 0.93)
Coping Score (2–13) - continuous	1.03 (0.96, 1.10)	0.98 (0.89, 1.07)	1.00 (0.94, 1.08)
Coping score level			
Low (0–5)	0.74 (0.47, 2.50)	1.05 (0.34, 3.23)	1.25 (0.40, 1.61)
Moderate (6–9)	1.16 (0.88, 1.53)	1.23 (0.84, 1.81)	1.25 (0.95, 1.66)
High (10–16) (ref)			
*Measures of Social Connectedness:*			
Feel safe in neighborhood because of positive/neutral relations within neighborhood	1.45 (0.74, 2.86)	1.81 (0.26, 1.25)	1.61 (0.72, 3.57)
Did NOT Know most of their neighbors and talk to them often	1.15 (0.81, 1.62)	0.70 (0.40, 1.23)	0.96 (0.67, 1.38)
Nearest person they would call in an emergency			
No one	0.54 (0.22, 1.32)	--	0.75 (0.21, 2.77)
Outside of neighborhood	0.66 (0.40, 1.08)	--	0.42 * (0.25, 0.68)
In the neighborhood (ref)			
Respondent/neighbor have NOT checked on each other during heat wave and/or called on each other in an emergency	0.51* (0.34, 0.76)	0.19 * (0.087, 0.42)	0.36 * (0.24, 0.54)
Social connectedness score: (0–3)			
0–1	1.15 (0.70, 1.87)	0.44 (0.19, 1.06)	0.84 (0.51, 1.39)
2	0.41 * (0.28, 0.59)	0.26 * (0.13, 0.49)	0.31 * (0.21, 0.46)
3 (ref)			

***** *p* < 0.05 compared to indicated reference.

**Table S5:** Association between the heat-related outcomes and measures of access to information (adjusted odds ratios and 95% confidence intervals).

	HRS	HRI	HRI + HRS
Age			
Young (<5)	0.13 ***** (0.04, 0.44)	--	0.44 ***** (0.26, 0.73)
Elderly (>60)	1.94 ***** (1.15, 3.29)	--	1.95 ***** (1.10, 3.45)
All other ages (5 ≤ age ≤ 60) (ref)			
Work location:			
Sun	2.20 ***** (1.29, 3.74)	--	1.82 ***** (1.08, 3.06)
Mixed	1.05 (0.62, 1.78)	--	1.12 (0.67, 1.87)
Shade			
Chronic preexisting condition	3.38 ***** (2.49, 4.60)	1.66 ***** (1.09, 2.51)	2.41 ***** (1.78, 3.26)
Diarrheal preexisting condition	--	3.42 ***** (1.78, 6.57)	--
Infectious preexisting condition	1.59 ***** (1.123, 2.24)	2.90 ***** (1.91, 4.43)	1.82 ***** (1.33, 2.49)
Main drinking water source:			
In-home tap	0.53 (0.27, 1.04)	--	--
Public (tap/bore hole)	0.44 ***** (0.20, 0.96)	--	--
From neighbor	0.83 (0.26, 2.62)	--	--
Purchased (bottled/50L jug) (ref)			
*Measures of Access to Information ^a^:*			
Visited a doctor for heat-related illness before	--	--	2.77 ***** (2.13, 3.57)
Heat warning source this past summer: People *vs.* Media	0.87 (0.61, 1.23)	0.82 (0.44, 1.52)	0.91 (0.64, 1.29)
Heat-related morbidity source where they have looked or would look			
Media	1.51 (0.87, 2.63)	1.54 (0.68, 3.54)	1.65 (0.92, 2.97)
Don't Know	1.86 (0.61, 5.65)	1.64 (0.23, 11.89)	1.60 (0.41, 6.19)
Community (ref)			
Person previously talked to about heat-related morbidity			
No one	0.92 (0.55, 1.55)	0.71 (0.36, 1.40)	0.70 (0.44, 1.12)
Community	1.43 (0.71, 2.85)	2.17 (0.87, 5.45)	1.76 (0.87, 3.57)
Professional (ref)			
NOT Worried about getting sick from heat	2.84 ***** (1.25, 6.45)	--	2.22 ***** (1.04, 4.75)
Had NOT sought heat-related morbidity info before	1.46 (0.62, 3.44)	11.18 ***** (2.75, 45.38)	2.56 ***** (1.20, 5.49)
*Social Connectedness Score ^a^:*			
0–1	2.87 ***** (1.41, 5.87)	0.86 (0.31, 2.41)	2.24 ***** (1.19, 4.22)
2	0.70 (0.44, 1.10)	0.40 ***** (0.21, 0.75)	0.71 (0.46, 1.07)
3 (ref)			

^a^ Covariates were tested in hypothesis; ***** *p* < 0.05 compared to indicated reference.
